# Local Serpin Treatment via Chitosan-Collagen Hydrogel after Spinal Cord Injury Reduces Tissue Damage and Improves Neurologic Function

**DOI:** 10.3390/jcm9041221

**Published:** 2020-04-23

**Authors:** Jacek M. Kwiecien, Liqiang Zhang, Jordan R. Yaron, Lauren N. Schutz, Christian J. Kwiecien-Delaney, Enkidia A. Awo, Michelle Burgin, Wojciech Dabrowski, Alexandra R. Lucas

**Affiliations:** 1 Department of Pathology and Molecular Medicine, McMaster University, Hamilton, ON L8S4L8, Canada; 2 Center for Personalized Diagnostics and Center for Immunotherapy, Vaccines and Virotherapy, Biodesign Institute, Arizona State University, Tempe, AZ 85287, USA; liqiang.zhang@asu.edu (L.Z.); jyaron@asu.edu (J.R.Y.); lschutz2@asu.edu (L.N.S.); eawo1@asu.edu (E.A.A.); mburgin@asu.edu (M.B.); 3 Faculty of Science, McMaster University, Hamilton, ON L8S4L8, Canada; ckdftw@gmail.com; 4 Department of Anaesthesiology and Intensive Therapy, Medical University of Lublin, 20-400 Lublin, Poland; w.dabrowski5@gmail.com

**Keywords:** spinal cord injury, inflammation, immune modulation, serpin, chitosan-collagen hydrogel

## Abstract

Spinal cord injury (SCI) results in massive secondary damage characterized by a prolonged inflammation with phagocytic macrophage invasion and tissue destruction. In prior work, sustained subdural infusion of anti-inflammatory compounds reduced neurological deficits and reduced pro-inflammatory cell invasion at the site of injury leading to improved outcomes. We hypothesized that implantation of a hydrogel loaded with an immune modulating biologic drug, Serp-1, for sustained delivery after crush-induced SCI would have an effective anti-inflammatory and neuroprotective effect. Rats with dorsal column SCI crush injury, implanted with physical chitosan-collagen hydrogels (CCH) had severe granulomatous infiltration at the site of the dorsal column injury, which accumulated excess edema at 28 days post-surgery. More pronounced neuroprotective changes were observed with high dose (100 µg/50 µL) Serp-1 CCH implanted rats, but not with low dose (10 µg/50 µL) Serp-1 CCH. Rats treated with Serp-1 CCH implants also had improved motor function up to 20 days with recovery of neurological deficits attributed to inhibition of inflammation-associated tissue damage. In contrast, prolonged low dose Serp-1 infusion with chitosan did not improve recovery. Intralesional implantation of hydrogel for sustained delivery of the Serp-1 immune modulating biologic offers a neuroprotective treatment of acute SCI.

## 1. Introduction

Spinal cord injury (SCI) causes prolonged morbidity and mortality with secondary physiological, economical, and psychological complications. It was estimated in 2016 that more than 27 million people worldwide have suffered long-term disability following SCI [1,2]. While many new experimental approaches are under investigation, there remains a large, unmet need for treatments to limit neurologic deficits caused by SCI.

SCI is characterized by extensive disruption of neurological tissue leading to a permanent loss of function. Progressive and persistent inflammatory responses after injury cause ongoing, prolonged and severe tissue destruction [3]. Early hemorrhage and necrosis are accompanied by acute inflammation with macrophage invasion, swelling and edema, and irreversible destruction forming a cavity of injury (COI) [3]. Macrophage counts markedly increase during the onset of the inflammatory phase on the third day after SCI, peak at one to four weeks, and subsequently gradually decline [3]. Macrophages are still present at 16 weeks after injury, albeit at low numbers [3]. Invasive macrophages contain luxol fast blue-positive granules of myelin and are exclusively CD68-positive and CD163-negative indicating a pro-inflammatory, destructive character [3].

Treatment for SCI with short term, intravenous methylprednisolone (steroid, NASCIS studies) [4,5,6,7,8,9,10], or steroid cocktails have been used, but with limited proven efficacy and have been associated with severe side effects [8,10,11,12,13]. Steroid use after SCI has remained hotly debated for decades [14,15]. Indeed, the American Association of Neurological Surgeons and the Congress of Neurological Surgeons published a medical evidence-based guideline with recommendation against the use of methylprednisolone in SCI [16]. Many minimally invasive and regenerative therapeutics are under investigation ranging from regenerative therapy with hydrogel implants, stem cells, growth factors, magnesium and antioxidants, among others, but without proven long term benefits at this time [1,2,4,5,6,7,11,12,13,17,18]. Chitosan hydrogels with or without growth factors have been proven to promote SCI restoration and axon regeneration in rat and monkey models [19,20,21].

Serine proteases in the thrombotic and thrombolytic cascades, in addition to driving clot formation and bleeding, also activate acute inflammatory responses [12,22,23,24,25,26,27]. Tissue- and urokinase-type plasminogen activators (tPA and uPA) are thrombolytic serine proteases which are released at sites of neuronal damage leading to hemorrhage and also activate matrix metalloproteinases (MMPs) increasing connective tissue degradation and enabling inflammatory cell invasion [22,23,24,25,26,28,29]. Serine proteases and metalloproteinases also activate and release membrane-bound chemokines and growth factors [22,23,24,25,26,28,29,30]. Serine protease inhibitors (serpins) are “suicide” inhibitors that have highly conserved structures and exist in all kingdoms. In mammals, serpins are highly prevalent, represent up to 2%–10% of the proteins in the circulating blood, and function to prevent excess bleeding or clotting [22,23,24,25,26,27,31,32,33]. Inhibition of serine proteases by serpins results in the covalent linkage of the serpin with target proteases which results in a serpin-enzyme complex (SEC) which is then taken up by SEC receptors such as the LDL receptor-related protein 1 expressed by neurons, hepatocytes, macrophages, and other tissues with subsequent metabolic breakdown of the receptor [34,35].

The role of serpins and serine proteases in SCI is complex and remains incompletely understood. Serpins, specifically neuroserpin (NSP) and plasminogen activator inhibitor-1 (PAI-1) are mammalian serpins that bind and inhibit tPA and uPA. NSP and PAI-1 have been assessed as treatments, as well as in the pathologic responses that occur after SCI. Treatment with exogenous NSP, which is given immediately after clamp compression-induced spinal cord injury, restores autophagic flux and improves functional outcomes by seven days post-injury in adult rats [27]. NSP-deficient mice have increased stroke volume as compared with wild type mice with normal NSP expression after cerebral artery occlusion [36]. In another study, spikes of increased PAI-1 expression (mRNA and protein) have been observed at mRNA and protein levels between one and three days post-injury in rats when subjected to balloon compression-induced SCI [37]. In the same study, treatment with exogenous tPA resulted in improved functional recovery when given one day post-injury by intraspinal injection. Conversely, tPA-deficient mice had reduced injury and improved functional recovery after contusion injury observed for six weeks [38]. Thus, modulation of serpins and their targets represent potential therapeutic targets in SCI but needs further study.

We recently reported that administration of the myxoma virus-derived immune modulating serpin, Serp-1, infused locally for seven days starting immediately after balloon crush SCI, reduced inflammation and improved functional recovery in rat models [13]. Serp-1 binds and inhibits tissue- and urokinase-type plasminogen activators (tPA and uPA, respectively), plasmin in the thrombolytic cascade, and thrombin and factor X in the coagulation/thrombotic cascade. This serpin protein biologic has proven efficacy for reducing vascular inflammation and improving mortality in a wide range of vascular injury, inflammation, and transplant models. Serp-1 was also tested in a Phase 2 clinical trial in patients with unstable coronary syndromes and coronary stent implants. In that study, the major adverse cardiac event (MACE) score was zero with no side effects observed, and there was a significant decrease in markers of heart damage [26]. Therefore, Serp-1 is a promising biologic for use in a variety of inflammatory conditions, including SCI.

We previously demonstrated that Serp-1 was effective after sustained subdural infusion for seven days to inhibit inflammatory infiltration following balloon crush SCI [13]. However, continuous infusion by indwelling catheters posed an increased risk for nosocomial infections and trauma with increased morbidity. Prior work in SCI models has also demonstrated that creating a “bridge” with an implanted biodegradable material, such as chitosan, can improve recovery after SCI [21] but other studies identified severe destructive inflammation initiated by the spinal crush injury as a major impediment in studying cellular and synthetic implants [21,39]. Therefore, addressing the post-SCI inflammation remains a fundamental issue in therapeutic strategies in neurotrauma. We recently demonstrated that Serp-1 maintained therapeutic efficacy to improve healing in a full-thickness wound mouse model when delivered by a chitosan-collagen hydrogel (CCH) [40]. Chitosan, in a hydrogel combination with the structural stability of collagen, produces a highly biodegradable material with excellent biocompatibility, low antigenicity, and prior success in animal models of SCI [41,42]. Here, we studied whether Serp-1 delivered by chitosan-collagen hydrogel implant provides therapeutic efficacy in a forceps crush-induced spinal cord injury model.

## 2. Experimental Section

### 2.1. Ethical Considerations

Experiments using male, 16 weeks old Long Evans rats, 370–410 g, were approved by the Animal Research Ethics Board at McMaster University (protocol AUP19-03-13) according to the Guides and Regulations of the Canadian Council of Animal Care.

### 2.2. Serp-1 Protein Expression and Purification

Recombinant Serp-1 (m008.1L, NCBI Gene ID# 932146) was expressed and secreted by a Chinese hamster ovary (CHO) cell line (Viron Therapeutics Inc., London, ON, Canada). GMP-compliant purification was performed with greater than 95% purity as determined by Coomassie-stained SDS-PAGE and reverse-phase HPLC. Serp-1 was endotoxin-free by LAL assay and stocked at −80 °C in 100 mM citrate buffer (pH 6.5) at a concentration of 1.8 mg/mL [26].

### 2.3. Preparation of Chitosan-Collagen Hydrogels

The procedure for preparing the chitosan-collagen hydrogel (CCH) was performed as previously described [42]. Briefly, 15 mg of low molecular weight chitosan (448869l, 75%-85% deacetylated, Sigma Life Science, St. Louis, MO, USA) was swelled in 10 mL of deionized water and gently rotated overnight at 4 °C. The excess water was removed by centrifugation at 1000 ×g for 15 min. The swollen chitosan product was frozen at −20 °C for 8 h followed by thawing overnight at 4 °C. The same volume of Serp-1 solution, containing 0, 0.1, or 1 mg protein, was added and the mixture was rotated at 4 °C for 8 h, and then frozen on dry ice and lyophilized overnight. Shortly before application, the lyophilized product was added to Type I collagen solution (C3867, Sigma Life Science, St. Louis, MO, USA) to a total volume of 500 µL to form a chitosan-collagen/Serp-1 gel at a Serp-1 concentration of 0, 10 μg (low dose), and 100 μg (high dose) per 50 μL gel, respectively. Previous characterization by our group demonstrated that this CCH material functions as a sustained release substrate, with in-tact Serp-1 continuing release after at least 4 days post-reconstitution [42]. Other groups have demonstrated similar chitosan-collagen hydrogel materials to release functional protein for 4 weeks [43].

### 2.4. Neurosurgery: Dorsal Column Fine Forceps Crush Model

The surgical procedure involved in the dorsal column fine forceps crush SCI has been previously described [44]. Briefly, 36 rats (Table 1) were induced for SCI surgery in 5% isoflurane in 95% oxygen and maintained under 4% isoflurane in 96% oxygen. A laminectomy was created at the T10 vertebrum, the dura was cut open, and the dorsal spinal column was crushed. Crushing was performed with jeweler’s forceps by inserting the forceps tips to a depth of 0.5 mm with tip spacing at 0.5 mm and pinching the tips shut for 10 s. Fifty microliters of chitosan-collagen hydrogel was injected gently into the crush lesion via a polished 21G needle. Then, the spinal muscles were closed with absorbable sutures and the skin with stainless steel staples.

For groups with subtherapeutic dose of Serp-1, stock protein was diluted in saline at 0.1 mg/mL and 2ML1 osmotic pumps (Alzet) were loaded with 2 mL of protein. To initiate subdural infusion, a small cut was created in the dura over the dorsal spinal cord into the area of the laminectomy and an intrathecal catheter was inserted over the cord cranially to approximate the catheter tip with the site of the injury. The other end of the catheter was connected to the pump and placed under the skin of the flank. The remainder of the procedure was the same.

Prior to waking from anesthetic, all rats received an injection of 0.4 mL ketoprofen analgesic (10 mg/mL, Anafen, Merial Canada, Inc., Baie d’Urfe, Quebec, Canada) and 5 mL of saline subcutaneously. Ketoprofen injections were repeated on days 1 and 2 post-surgery and rats with hemorrhagic cystitis were treated with an intramuscular injection of 50 µL enrofloxacin (50 mg/mL, Baytril^®^, Mississauga, Canada) for 3–5 days until blood cleared from the urine.

### 2.5. Post-Surgical Period

Rats with hind end paralysis, with urinary bladder distended with hemorrhagic urine, moderate to marked dehydration, lethargy and reduced body temperature, or extensive self-inflicted trauma, together defined as endpoint criteria, were humanely euthanized prior to the completion of the study, as indicated in the footnote of Table 1 (*N =* 4) [45].

### 2.6. Neurological Tests

A locomotor test derived from components of the Bassso, Beattie, Bresnaham (BBB) [46] and Modified Tarlov tests [47] was used for assessing motor function after SCI. Locomotor tests using modifications of the BBB testing have been used previously as the BBB test was developed to assess spontaneous recovery from SCI and may not be ideally applied to treatments which result in recovery that deviates from the scale [48]. The addition of supplemental behavioral analyses have been reported to improve the accuracy of assessments from locomotor tests alone [49]. Therefore, we have additionally supplemented the hind end motor function tests with the addition of a toe-pinch withdrawal analysis to assess the pain sensation and the strength of the hind limb withdrawal, as well as urinary bladder dysfunction scoring. The motor function of the hind limbs was observed and scored after SCI once daily in freely moving rats in a cage and scored as described in Table 2. The hind leg toe-pinch withdrawal response was scored separately for the left and right legs (Table 3). Urinary bladder function was recorded daily after SCI following the scoring standard as listed in Table 4. Rats with bladder distension were voided manually 1-2 times per day until bladder function returned during the second week post-SCI. Body weight of the experimental rats was taken every 3rd day post-SCI.

### 2.7. H&E and Immunohistochemistry

At day 7 and 28 post-SCI, the rats were overdosed with sodium pentobarbital, 80 mg/kg body weight and whole-body perfusion performed with buffered formalin [45]. The spine, together with the spinal cord, was removed and post-fixed in formalin for 24 h, then placed in a decalcifying solution; formalin supplemented with 8% EDTA, pH 7.2, placed on a rotating shaker. Decalcifying solution was replaced twice a week and after 3-4 weeks, the bones of the spine were soft. A 2.5 cm long segment of the spine including the laminectomy from each rat was cut in the sagittal orientation, processed in rising concentrations of ethyl alcohol and xylene, and embedded in paraffin wax.

For the histologic analysis, 5 µm thick longitudinal sections were mounted on glass slides, processed for IHC and coverslipped. Sections were additionally stained by immunohistochemistry (IHC) for CD3 (Abcam, #ab5960, 1:100), GFAP (Proteintech, #16825-1-AP, 1:200), neurofilament (NF-M) (DSHB, #2H3, 1:50), cleaved caspase-3 (Asp175) (Cell Signaling, #9661, 1:200), and F4/80 (Abcam, # ab100790, 1:100).

### 2.8. Pathology Imaging and Analysis

The sections of the spinal cord were analyzed under a Nikon Eclipse 50i or Olympus BX51 light microscope and photographed at 2–60× magnification. Cell counting and morphometric analysis was performed in ImageJ/FIJI. Cell counts of longitudinal sections at a similar depth in each animal were performed blinded on 3–6 peri-injury fields per rat. After counting, the rats were unblinded and counts from each group were averaged. Statistics were performed on the averages of each group. Evidence of astrogliosis and containment (coalescence of GFAP+ cells) of the injury site was scored blinded according to the metrics described in Table 5 and representative fields are given in Figure S1.

### 2.9. Statistics

Data were analyzed in GraphPad Prism v8.1.2 by T-test and two-way Analysis of Variance (ANOVA) with Fisher’s Least Significant Difference (LSD) post hoc analysis. Significance indicated by * *p* < 0.05, ** *p* < 0.01, *** *p* < 0.001, and **** *p* < 0.0001.

## 3. Results

### 3.1. Treatment with Serp-1 Loaded into Chitosan-Collagen Hydrogel Improves Clinical Scores in SCI Rats

**Figure 1:**
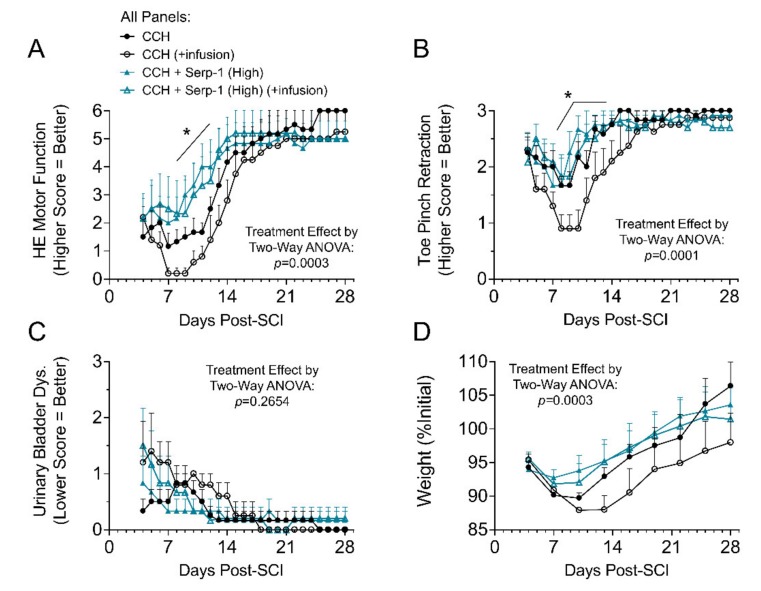
Clinical evaluation of rats after spinal cord injury (SCI) treated. (**A**) Hind end (HE) motor function scores for SCI rats treated with 50 µL chitosan-collagen gel only (chitosan) or 50 µL gel with 100 µg Serp-1 (chitosan-Serp-1 high). Higher score indicates better function; (**B**) Average toe pinch retraction scores from both left and right toes. Higher score indicates better function; (**C**) Urinary bladder dysfunction scores. Lower score indicates better function; (**D**) Weight tracking of rats post SCI, normalized to initial weights on the day of injury. Statistics calculated by two-way ANOVA with Fisher’s LSD of Serp-1 chitosan-collagen hydrogels (CCH) vs. CCH both with infusion. The main treatment effect is listed on the figure, while specific day significance is indicated by an asterisk with bar where * *p* < 0.05. Mean and standard error indicated. Legend given in panel A is identical for all panels.

In prior work, we found that high dose Serp-1 infusion (1 mg/week) was therapeutically effective, while low dose Serp-1 infusion (0.2 mg/week) was not beneficial after balloon crush SCI in rats [13]. The macrophage counts in the cavity of injury, however, were not different between these two doses of Serp-1 [13]. We tested implantation of chitosan-collagen hydrogel loaded with low dose (10 µg in hydrogel) and high dose (100 µg in hydrogel) Serp-1 alone and, additionally, with a low, subtherapeutic infusion of Serp-1 (0.2 mg/week infused) for comparison to our prior work. In this study, the functional recovery after high dose Serp-1 delivered by chitosan-collagen hydrogel was significantly improved at days seven to fourteen, but prolonged additional low dose Serp-1 infusion did not further improve outcomes, potentially due to secondary injury caused by catheter implant together with a protracted infusion. The hind limb motor function was recorded and scored from day three to 28 days post SCI (Table 1 and Figure 1A). Implantation of the chitosan-collagen hydrogel loaded with high dose of Serp-1 (100 µg in 50 µL hydrogel) into the dorsal column crush site resulted in less pronounced neurologic deficit and significantly faster recovery of motor function within the first 21 days as compared with scores of neurological deficits in SCI rats implanted with gel only (*p* = 0.0003). The ability to statistically discriminate between conditions was diminished after the first 21 days. Toe pinch withdrawal scores in high dose Serp-1 CCH treated rats were less pronounced in the first week and returned towards normal values with strong withdrawal during the second week post SCI as compared with CCH alone and low dose Serp-1 CCH (Figure 1B, *p* = 0.0001). Urinary bladder dysfunction did not significantly improve earlier in rats treated with high dose Serp-1 in the CCH versus rats with CCH alone (Figure 1C, *p* = 0.2654). Post-surgical weight loss (Figure 1D) was limited in rats treated with high dose Serp-1 CCH (*p* = 0.0003). Low dose Serp-1 CCH (10 µg in 50 µL hydrogel) achieved subtherapeutic dosing and did not improve hind-end motor function, toe pinch retraction, or urinary bladder dysfunction scores. Data traces for low dose Serp-1 CCH are given in Figure S2. To understand the specific biological effects of Serp-1 on the healing process after SCI, we focused on high dose Serp-1 without infusion treatment in subsequent analyses.

### 3.2. Treatment with Chitosan-Collagen Hydrogel with Serp-1 Reduces the Extent of Spinal Cord Damage

**Figure 2:**
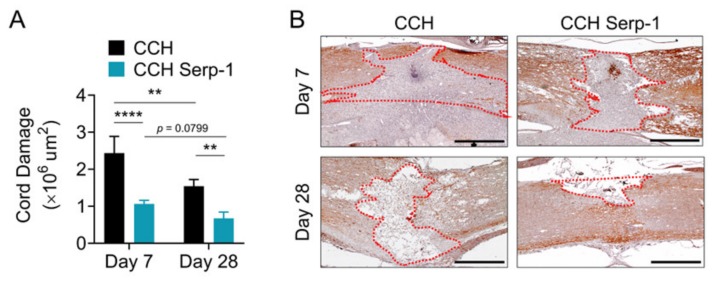
Serp-1 delivered by chitosan-collagen hydrogel reduces cord damage in rat SCI. (**A**) Quantification of cord damage area as measured by loss of neurofilament (NF-M) staining for post-SCI treated with 50 µL chitosan-collagen hydrogel alone or 50 µL chitosan-collagen hydrogel with 100 µg Serp-1 at days 7 and 28 after injury. Mean and standard error are presented. Statistics performed by two-way Analysis of Variance (ANOVA) with Fisher’s Least Significant Difference (LSD) post hoc where ** *p* < 0.01 and *** *p* < 0.0001. (**B**) Representative immunohistochemical staining (brown) of NF-M of conditions quantified in panel A. Dotted red area illustrates representative examples of quantified NF-M-negative areas. Boundaries defined are based on continuous tissue outside of the injury area and do not include excessive “spill over” which extend beyond the normal spinal cord boundaries (e.g., day 7 chitosan). Scale bars are 500 µm.

The area of the crush of the dorsal column was infiltrated by a severe granulomatous infiltration that completely obliterated the spinal cord and resulted in apparent extension of the size of the crush by weeks one and four post-injury, consistent with previous histologic studies using the dorsal column crush model [50,51] and the epidural balloon crush model [13]. The area of neuronal loss (obliteration) of the normal spinal cord structure was assessed by immunohistochemical staining for neurofilament medium polypeptide (NF-M) (Figure 2). Analysis of the cord damage area at seven days post-injury indicated that high dose Serp-1 delivered by chitosan-collagen hydrogel significantly limited the extent of obliteration versus chitosan-collagen hydrogel alone (*p* < 0.0001). The area of cord obliteration was reduced at 28 days for rats with SCI after the implant of hydrogel alone (*p* = 0.0037) or for hydrogel with Serp-1 (*p* = 0.0799). At day 28 post-injury, the implant of hydrogel with Serp-1 significantly reduced cord damage as compared with CCH alone (*p* = 0.0061). We observed a similar limitation of injury on staining with hematoxylin and eosin (not shown). Thus, high dose Serp-1 limits the area of SCI damage when delivered by chitosan-collagen hydrogel after dorsal column crush injury.

### 3.3. Treatment with Chitosan-Collagen Hydrogel Containing Serp-1 Reduces Apoptosis in Rat Spinal Cord after SCI

**Figure 3:**
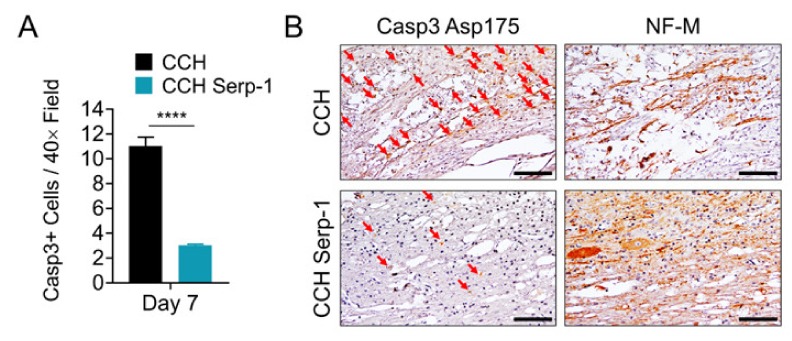
Serp-1 delivered by chitosan-collagen hydrogel reduces apoptosis in rat SCI. (**A**) Quantification of Casp3+ cells in areas defined as NF-M positive in serial spinal cord sections of rats post-SCI treated with 50 µL chitosan-collagen hydrogel alone or 50 µL chitosan-collagen hydrogel with 100 µg Serp-1 at days 7 after injury. Mean and standard error are presented. Statistics performed by T-test where **** *p* < 0.0001. (**B**) Representative immunohistochemical staining (brown) of cleaved, active Casp3 (Asp175) and NF-M in serial sections of the same conditions quantified in panel A. Red arrows indicate cells positive for active Casp3. Scale bars are 50 µm.

Next, we examined the potential mechanisms by which Serp-1 CCH treatment limits inflammatory cord damage after dorsal column crush. We performed immunohistochemical staining for active caspase-3 (Casp3, cleaved at Asp175) and quantified the number of Casp3-positive cells in the local damage area and specifically the association with NF-M-positive areas on the serial section (Figure 3). Rats treated with chitosan-collagen hydrogel alone had significantly more Casp3-positive cells in areas of positive NF-M staining at day seven post-injury versus rats treated with hydrogel containing high dose Serp-1 (*p* < 0.0001). Consistent with prior reports that apoptotic cells peak within a week of SCI and decrease over time, we found no substantial evidence of Casp3-positive cell staining at day 28 post-injury [52]. Thus, the limitation of neural injury by high dose Serp-1 after SCI in rats is associated with a reduction in apoptosis during the early phase of injury.

### 3.4. Treatment with Chitosan-Collagen Hydrogel with Serp-1 Reduces T Cell Infiltration in Spinal Cord in SCI Rats

**Figure 4:**
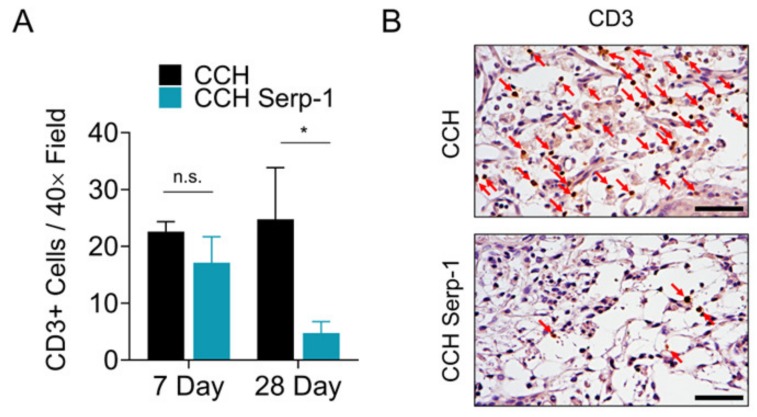
Serp-1 delivered by chitosan-collagen hydrogel decreases T-cell invasion in rat SCI. (**A**) Quantification of CD3+ cells in the area of injury in spinal cords of rats post-SCI treated with 50 µL chitosan-collagen hydrogel alone or 50 µL chitosan-collagen hydrogel with 100 µg Serp-1 at days 7 and 28 after injury. Mean and standard error are presented. Statistics performed by Two-Way ANOVA with Fisher’s LSD post-hoc where * *p* < 0.05 and n.s. is not significant. (**B**) Representative immunohistochemical staining (brown) of CD3+ cells at day 28 of the same conditions quantified in panel A. Red arrows indicate cells positive for active CD3. Scale bars are 25 µm.

Prior work from other groups indicated that treatments resulting in improved recovery after SCI were associated with a reduction in cluster of differentiation 3 (CD3)-positive T cells in the spinal cord [53,54]. Thus, we investigated whether Serp-1 CCH reduced T cell invasion in the spinal cord after SCI (Figure 4). Immunohistochemical staining for CD3-positive T cells in the area of injury indicated there was no significant difference between CCH controls and high dose Serp-1 CCH at seven days post-injury (*p* = 0.4089), whereas treatment with Serp-1 did significantly reduce CD3-positive cells in the area of injury by 28 days post-injury (*p* = 0.0293). We did not observe any effect on F4/80 macrophages in the same areas of analysis (Figure S3).

### 3.5. Treatment with Chitosan-Collagen Hydrogel with Serp-1 Promotes Earlier Astrogliosis in the Rat Spinal Cord after SCI

**Figure 5:**
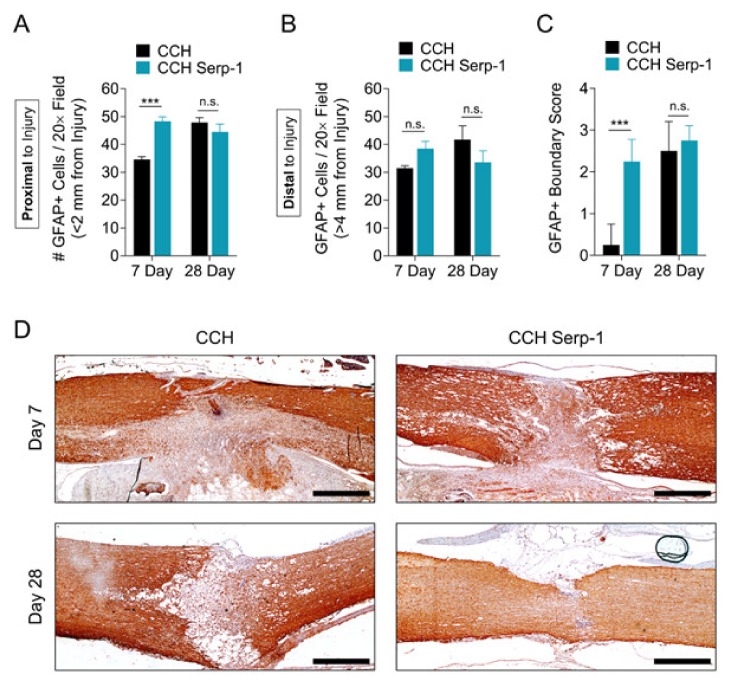
Serp-1 delivered by chitosan-collagen hydrogel stimulates protective astrogliosis in rat SCI. (**A, B**) GFAP+ cell counts in fields (A) proximal (<2 mm) or (B) distal to the site of injury (>4 mm) in spinal cords of rats treated with 50 µL chitosan-collagen hydrogel alone or 50 µL chitosan-collagen hydrogel with 100 µg Serp-1 at days 7 and 28 after injury. (**C**) Scoring of GFAP+ boundaries around the area of injury in spinal cords of rats post-SCI treated as in panels A and B. Mean and standard error are presented. Statistics performed by Two-Way ANOVA with Fisher’s LSD post-hoc where *** *p* < 0.001 and n.s. is not significant. (**D**) Representative immunohistochemical staining (brown) of GFAP+ cells of the same conditions quantified in panel A. Scale bars are 500 µm.

While early assessments of astrogliosis in the spinal cord were thought to be detrimental in SCI, in the past decade, an alternate protective role for astrocyte activation and compartmentalization of traumatic damage has been identified as a proposed mechanism to limit inflammatory exacerbation [3,50,55,56,57]. We sought to determine the effect of Serp-1 treatment on glial fibrillary acidic protein (GFAP)-positive astrocytes and astrogliosis in the spinal cord after SCI in our model (Figure 5). The number of GFAP+ cells in areas proximal to the injury (<2 mm) was significantly increased (*p* = 0.0001) at day seven in rats treated with Serp-1 CCH versus CCH alone (Figure 5A). Both Serp-1 and CCH had similar levels of GFAP+ cells proximal to the injury by day 28. This induced GFAP+ response was not observed at sites distal to the injury (>4 mm) (Figure 5B). We further analyzed the phenotype of the GFAP response in this model. On the basis of the well-defined mechanism and phenotype of astrocyte hypertrophy and coalescence at the border of spinal lesions during astrogliosis [56], we defined a scoring system for characterizing the degree of GFAP-positive compartmentalization (Table 5). Histological scoring indicated that Serp-1 CCH stimulated an earlier astrogliosis and GFAP-positive compartmentalization of spinal cord lesions at sseven days post-injury versus chitosan-collagen hydrogel alone (*p* = 0.0001), whereas the degree of compartmentalization was equal between groups at 28 days post-injury (*p* = 0.6438). As expected, the GFAP+ cell staining was most intense nearest to the site of injury, with cells distal to the injury remaining small and fewer in number (Figure S4). Taken together with the limitation of neural injury as measured by NF-M staining (Figure 2) and improved functional recovery (Figure 1), these results suggest that the early astrogliosis, as measured here by the total number of GFAP cells in proximity to the injury and by boundary scoring, was stimulated by Serp-1 CCH and was protective after dorsal column crush-induced SCI. While the GFAP scoring, here, was performed blinded, further work on three-dimensional analysis of GFAP containment would be helpful to more completely understand this potentially important phenomenon.

## 4. Discussion and Conclusions

After traumatic SCI, there is extensive damage to the spinal cord structural architecture in the spinal cord, initiating a complex secondary damage, which includes inflammation with death of neurons and glial cells. Early investigations reported that immune suppression with steroids was an effective treatment option, but steroid use after SCI has been abandoned due to a lack of proven benefit [16]. Current clinical management of spinal cord injuries focuses on limiting negative effects on quality of life (e.g., pain), reducing psychological impacts, and development of technological and mechanical solutions for recovering mobility and function. Thus, there remains a large, unmet need for pharmacologic interventions to improve clinical outcomes of SCI.

Delivery of therapeutic agents directly to the spinal cord after SCI is an increasingly popular experimental approach for testing new treatments. Here, we chose a chitosan-collagen hydrogel as a carrier for our immune modulating protein, Serp-1. The chitosan-collagen carrier was chosen based on well-characterized properties of tunable structural stability, excellent biocompatibility, low antigenicity, and high biodegradability [43,58,59]. Other researchers have already demonstrated that chitosan-collagen carriers can deliver therapeutic agents to the spinal cord [21,41]. In prior work neurotrophin-3 (NT3), a neurotrophic growth factor that promotes proliferation of neural stem cells, enhanced functional recovery after SCI in rats when delivered by chitosan-collagen carriers [21,41]. We recently reported that Serp-1 maintains function when released from chitosan-collagen in vivo in a full-thickness wound healing model [42]. Thus, we pursued this mode of delivery for SCI treatment in our model.

Recently, we determined that SCI initiates a severe, destructive, and extraordinarily protracted inflammation characterized by infiltration of necrotic sites by phagocytic, CD68+/CD163- macrophages [3]. We previously reported that treatment with this myxoma virus-derived Serp-1 biologic, functions as a therapeutic immune modulator in a wide array of mammalian models of disease, with improved functional recovery in rats after balloon crush spinal cord injury when given as a high-dose continuous subdural infusion [13]. Here, we assessed whether Serp-1 can maintain therapeutic efficacy after dorsal column forceps crush-induced SCI when delivered in a biodegradable chitosan-collagen hydrogel. We found that when given at a therapeutic dose in a single administration to the injured site, Serp-1 stimulated early functional recovery as assessed by locomotor analysis of hind end motor function together with toe pinch retraction and urinary bladder dysfunction scoring, while avoiding toxic weight loss after injury.

In prior work, we note that we found functional recovery from SCI with Serp-1 delivered by an osmotic infusion pump with catheter placement in the spinal cord [3]. Here, we tested whether Serp-1 CCH was effective with or without additional pump and catheter placement to assess the effect of this secondary perturbation. We note that high dose Serp-1 CCH consistently promoted functional recovery after forceps crush SCI irrespective of additional catheter-induced trauma, while CCH-only controls had worsened outcomes when there was secondary trauma by catheter insertion. Thus, high dose Serp-1 CCH is sufficient as a standalone therapeutic treatment in this model.

We performed histopathologic analysis of the spinal cords of rats treated with chitosan-collagen hydrogel alone or hydrogel with the higher, therapeutic dose of Serp-1 to investigate the mechanism by which the outcome was improved after SCI. We found that the area of neural damage, as assessed by NF-M staining, was limited in rats treated with CCH Serp-1. On the basis of the serial section staining, the areas of NF-M staining displayed reduced apoptosis when treated with Serp-1, thus, the limitation of injury was possibly mediated by suppression of apoptosis of glial and neuronal cells. Previous reports have demonstrated that inhibition of apoptosis via the caspase-3 inhibitor DEVD results in improved functional recovery and limited damage area in rats after contusion-induced acute SCI [60].

Serp-1 has previously been found to modulate infiltration of both T cells [24,61] and macrophages [3,22,42], dependent on the preclinical model being studied. Immunologically, we found in the current study that CD3-positive T cells, but not macrophages, were reduced more quickly in the area of injury after treatment with Serp-1. Others have reported that treatments which improve functional recovery can also suppress T cell infiltration in the spinal cord after injury [53]. T cells express urokinase-type plasminogen activator receptor (uPAR) [62] and immune cells positive for uPAR are known to infiltrate neural tissue after traumatic injury [63]. Serp-1 binds to and acts via uPAR [24,42]. Thus, it is possible that Serp-1 either prevents sustained T cell invasion and activation, or promotes resolution, via binding to uPAR. Further studies are needed to further characterize specific T cell phenotype responses in the area of injury [64] to better understand this Serp-1-mediated effect.

We also found that in addition to reducing CD3 T cells, Serp-1 treatment promoted an earlier astrogliosis resulting in compartmentalization of the area of injury. It is now well described that astrogliosis has a dual role in spinal cord injury with pathologic effects on neurogenesis, as well as a protective effect that limits the expansion of inflammation and damage [3,55,56,57]. Compartmentalization of the cavity of injury post-SCI, coincides with suppression of release of pro-inflammatory and pro-apoptotic factors, causing a secondary suppression of immune cell infiltration [3]. Consequently, astrogliosis promoted by Serp-1 can cause an upstream limitation of injury via sequestration of products produced during the acute injury, suppressing inflammatory infiltration and reducing apoptosis [65]. Spinal cord injury is associated with a compartmentalization mediated by reactive astrocyte tight junctions containing claudin 1 (CLDN1), CDLN4 and junctional adhesion molecule A (JAM-A) [55]. Additional studies have identified transcriptional regulation of this process by signal transducer and activator of transcription 3 (STAT3) [56,57]. Whether the glial scar eventually degrades, or whether accessory nerve transduction paths develop, is a topic of intense investigation.

We note that all groups trend towards functional improvement over time in the rat crush model, but early, significant improvement occurred when Serp-1 was given at a 100 µg dose in CCH (which we refer to as a “high” dose in this study), indicating an early therapeutic benefit of this immune modulating biologic in the rat SCI model. Small animal (e.g., rats and mice) models of spinal cord injury do have limitations as compared with larger models (e.g., pigs and dogs) for modeling of human disease. There is ongoing discussion about the role of differences in gray matter volume and reinnervation, axon length, and subtle inherent differences in immune system responses [66]. These limitations aside, the rat model is the most tractable preclinical system for assessing functional and histologic recovery in traumatic SCI. It needs to be noted, however, that the neurological tests used in the present study and the BBB locomotor test used by others are affected and potentially limited by two general problems. Firstly, in untreated groups of rats the scores improve over the course of the first four weeks post SCI, despite the severity of inflammation and associated destruction of the spinal cord tissue and persistence of peri-lesional edema [3]. Secondly, the scores of neurological tests stabilize by the week 4 post-SCI and do not change after that despite the continuous destructive inflammation and peri-lesional edema for at least three months longer [3]. Therefore, there is no apparent correlation between the improvement in neurological scores and the pathology of SCI and neurological tests. Thus, there remains a gap in our current understanding of ongoing immune mediated damage after SCI and the capacity for neurological functional improvement further complicating interpretation.

In summary we report an early observed benefit for a local serpin hydrogel implant after SCI in a rat crush model. Further study with longer term delivery, as well as studies in larger animal systems, or “Phase 0” clinical trials in humans are necessary to more directly assess the translational potential for this promising biologic as a therapy for spinal cord injury.

## Figures and Tables

**Table 1:** Numbers of rats used in this study.

Treatment	Infusion	Days Follow-Up	Number of Rats
Chitosan hydrogel	No	7	6
Chitosan-Serp-1 low (10 µg)	No	7	6
Chitosan-Serp-1 high (100 µg)	No	7	6
Chitosan hydrogel	No	28	7 ^a^
Chitosan-Serp-1 low (10 µg)	No	28	6 ^b^
Chitosan-Serp-1 high (100 µg)	No	28	6
Chitosan hydrogel	Yes	28	5 ^c^
Chitosan-Serp-1 low (10 µg)	Yes	28	5 ^d^
Chitosan-Serp-1 high (100 µg)	Yes	28	6

^a^One rat euthanized on day 23 post-SCI due to severe skin lesions. ^b^ One rat found dead on day 20 post-SCI. ^c,d^ One rat euthanized in indicated groups on day 10 post-SCI due to self-inflicted trauma.

**Table 2:** Hind end (HE) motor function score standard.

Score	Description
0	Both hind legs have no motion.
1	One hind leg has flexing motion caudal to the level of the hip joint, with the plantar surface of the foot up, no weight support.
2	Both legs have flexing motion caudal to the hip, with the plantar surface of the foot up, no weight support or one leg has flexing motion beyond the hip, no body support, the other leg has no motion.
3	One leg has flexing motion beyond the hip, with the dorsal surface of the foot up, no weight support, the other leg has flexing motion caudal to the hip, with the plantar surface of the foot up; or one leg has flexing motion beyond the hip, with dorsal surface of the foot up, with body weight support but the other leg has no motion.
4	Both legs have flexing motion beyond the hip, with the dorsal surface of the foot up, but no body weight support; or one leg with the flexing motion beyond the hip with body support and the other leg with flexing motion caudal to the hip, with the plantar surface of the foot up, but no body weight support.
5	One leg has flexing motion beyond the hip with body weight support, the other leg flexing motion beyond the hip, with the dorsal surface of the foot up, but no body support.
6	Normal gait, no apparent weakness or proprioceptive deficits.

**Table 3:** Hind limb toe-pinch retraction response score standard.

Score	Description
0	No toe retraction
1	Weak, no jerking
2	Weak with jerking
3	Strong/normal with jerking

**Table 4:** Urinary bladder dysfunction score standard.

Score	Description
3	Distended with hemorrhagic urine (treated with 50 µL Baytril s.i.d I/M until clear)
2	Distended with some blood in urine at the beginning of voiding
1	Distended, clear urine
0	Normal function, not distended

**Table 5:** Degree of containment scoring.

Score	Description
0	No containment
1	Weak containment, <1 side of the injury
2	Mild containment, >1 and <2 sides of the injury
3	Moderate containment, >2 sides of the injury
4	Complete containment, all sides of the injury

## Supplementary Materials

The following are available online at https://www.mdpi.com/2077-0383/9/4/1221/s1, Figure S1: Representative images of GFAP scoring used in blinded analysis, Figure S2: Functional analysis of all experimental groups, with and without subtherapeutic dose of Serp-1 by infusion, Figure S3: Serp-1 delivered by chitosan-collagen hydrogel does not affect F4/80 macrophages in rat SCI, Figure S4: Assessment of GFAP+ cell activation with proximity to injury site by immunohistochemistry.
