# Supplementary material for: Local Serpin Treatment via Chitosan-Collagen Hydrogel after Spinal Cord Injury Reduces Tissue Damage and Improves Neurologic Function
Source: J Clin Med. 2020 Apr 23;9(4):1221. doi: 10.3390/jcm9041221 (PMC7230793; doi:10.3390/jcm9041221)
Supplement: Supplementary file 1 [file jcm-09-01221-s001.pdf]

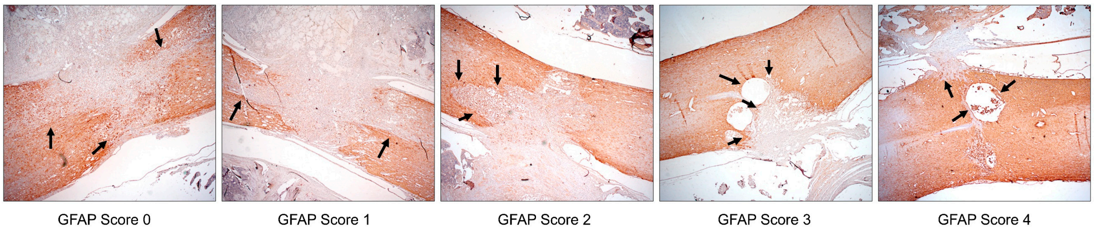

Figure S1. Representative images of GFAP scoring used in blinded analysis. Black arrows identify lack of containment in Score 0 and key areas of coalescence/containment in Scores 1-4.

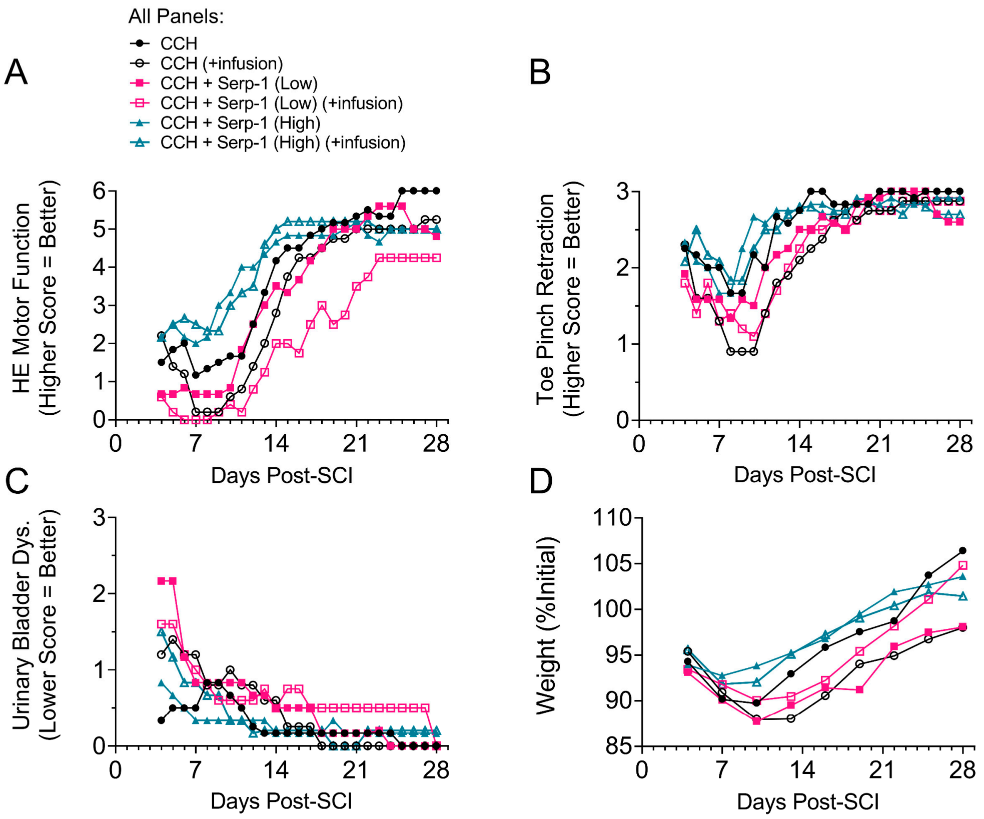

Figure S2. Functional analysis of all experimental groups, with and without sub-therapeutic dose of Serp-1 by infusion. Only means are shown for clarity.

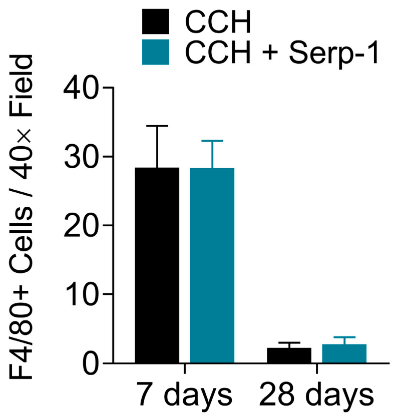

Figure S3. Serp-1 delivered by chitosan-collagen hydrogel does not affect F4/80 macrophages in rat SCI. Mean and standard error of quantification of F4/80+ cells in the area of injury in spinal cords of rats post-SCI treated with 50 μL chitosan-collagen hydrogel alone or 50 μL chitosan-collagen hydrogel with 100 μg Serp-1 at days 7 and 28 after injury. Statistics performed by Two-Way ANOVA with Fisher's LSD post-hoc.

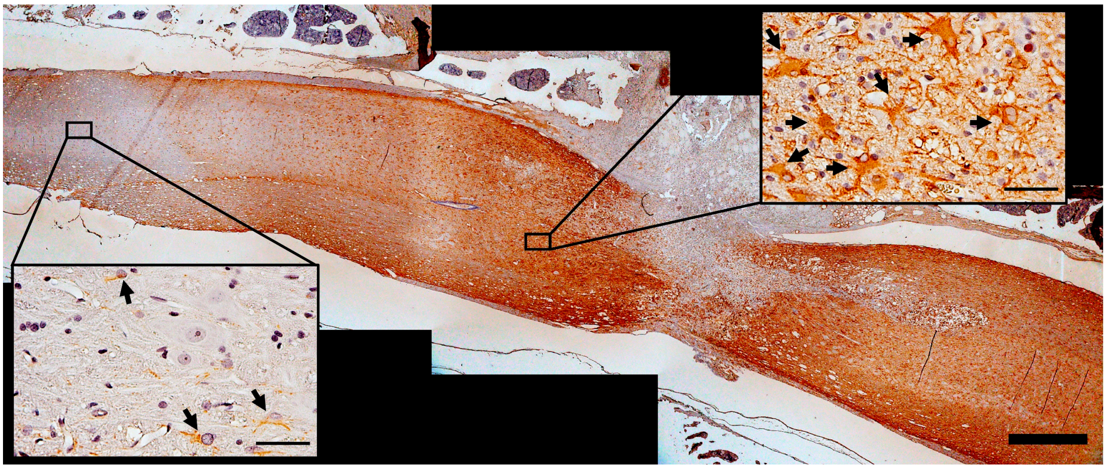

Figure S4. Assessment of GFAP+ cell activation with proximity to injury site by immunohistochemistry. Cells nearest to the injury (top right inset) display hypertrophic activation and are greater in number, while cells distal to the injury site (lower left inset) are small and fewer in number. Representative cells are indicated by arrows. Scale bars are 1000 μm for the large, stitched image and 50 μm for the insets.
